# Stress field disruption allows gas-driven microdeformation in bentonite to be quantified

**DOI:** 10.1038/s41598-023-41238-7

**Published:** 2024-01-08

**Authors:** Caroline C. Graham, Jon F. Harrington

**Affiliations:** British Geological Survey, Nicker Hill, Keyworth, Nottinghamshire NG12 5GG UK

**Keywords:** Civil engineering, Environmental impact, Seismology

## Abstract

Geological disposal of radioactive waste is being planned by many countries. Bentonite clay is often included in facility design, providing a barrier to radionuclide migration. Gas, generated by the waste or corrosion of waste canisters, may disrupt the properties of the bentonite. Robust prediction of this interaction is, therefore, necessary to demonstrate safe facility evolution. In some cases, gas may deform the clay, resulting in localised flow; however, the nature of this deformation has been widely debated. Accurate numerical representation of this behaviour has been limited by a shortage of information on the degree/distribution of deformation. Using experimental data from gas injection tests in bentonite, we show that first order fluctuations in the stress field can provide this information. We show that hundreds of microdeformation events can be detected, with similar characteristics to established fracturing phenomena, including earthquakes and acoustic emissions. We also demonstrate that stress field disruption (i) is spatially localised and (ii) has characteristics consistent with gas pathway ‘opening’ and ‘closure’ as gas enters and exits the clay, respectively. This new methodology offers fundamental insight and a new opportunity to parameterise and constrain gas advection models in clays and shales, substantially improving our capacity for safe facility design.

## Introduction

Permanent burial of radioactive waste in the subsurface is the preferred concept for safe disposal in multiple countries around the world^1,2^. The type of host rock surrounding a Geological Disposal Facility (GDF) will be dependent on each national concept^1–3^, but a common feature is an Engineered Barrier System (EBS), surrounding the waste canister and sealing boreholes and shafts after closure^4^. Bentonite clays are often incorporated for this purpose, particularly in the context of high-level waste facilities^4^. Over the lifetime of a GDF, gases may be generated by the waste itself, corrosion of the waste canisters and microbial action^1^. Hydrogen, in particular, is a likely product, although other gases may also be relevant, depending on the nature of the waste. Evaluation and management of the interaction between these gases and the EBS is, therefore, required to assess repository safety^4^.

Bentonite clays are rich in smectite, a phyllosilicate mineral with the capacity to absorb water and swell^5^. Very low hydraulic permeabilities, a relatively deformable matrix and a low tensile strength are key characteristics of these materials, which strongly influence their gas flow properties^6,7^. Gas migration in clays can occur by 4 primary mechanisms^7^: (i) dissolution of gas into pore water (governed by Henry’s Law) and diffusion (governed by Fick’s law), (ii) advection after overwhelming pore network capillary forces, resulting in the displacement of water through pre-existing porosity, (iii) advection through the creation of new voidage, via deformation of the matrix, or (iv) macroscale hydrofracture. The first of these mechanisms will always occur, but if the diffusion of gas is not sufficient to match its generation rate, then pressure will build until advection (ii or iii) eventually occurs. Which advection mechanism will dominate depends on whether the pore throat radii are sufficiently large to allow water displacement at pressures below those necessary to deform the clay matrix^7^. Lower clay contents and lower saturation states (which are expected to vary during repository lifetime) are thought to favour the former, whilst higher clay content and higher saturations may increase the likelihood of deformation^4,8^, although the conditions for this transition and the understanding necessary to select the correct conceptual model remain poorly constrained^8,9^.

Multiple experimental programmes document the occurrence of gas flow in clays, above a critical threshold^8,10–17^, which has been shown to relate to the internal stress state of the clay, σ_ij_,^13,15,16^. This behaviour has been attributed to gas advection via deformation of the matrix (iii), also termed ‘pathway dilatancy’. In such cases, gas migration is accompanied by complex hydromechanical coupling, which has proven challenging but necessary for comprehensive numerical simulation of the process^9,18^. Nevertheless, insufficient information is available to populate gas flow models incorporating these features (e.g., spatial and temporal distribution of pathways), limiting the development of predictive simulations in this field^18,19^.

This information remains limited for clay-rich materials because: (i) the narrow pore throats^20^ and gas pathway apertures involved (> 50nm,^21^) hinder the use of conventional analytical and imaging methodologies on a representative scale^22,23^, (ii) gas pathways close on depressurisation and cannot be distinguished from damage using standard petrological/analytical techniques^21^, (iii) clay-rich materials are highly attenuating to acoustic energy^24^, limiting the success of conventional approaches, such as the monitoring of acoustic emissions to distinguish deformation mechanisms and locations. Recent attempts to image gas pathways in natural clay using microCT analysis may provide an insight into the distribution of residual pathways after gas injection^25^. However, the large scale of these features and their persistence after testing is inconsistent with previous observations^21^ and is suggestive of permanent sample damage, potentially due to desaturation or depressurisation of gas after testing. Such post-test methodologies are also limited in that they do not provide real-time information relating to pathway development under pressurised conditions and are likely to detect only the largest features.

In this study, we show that small perturbations in monitored stresses, during gas injection testing of bentonite clay, bear similar characteristics to earthquakes and acoustic emissions generated during rock fracture. Source analysis of these events demonstrates for the first time that gas advection in this context is concurrent with the opening of new voidage within the clay. We show that the cumulative number of detected events provides a powerful tool to assess deformation development during gas breakthrough and the first evidence of its spatial evolution. This represents the first truly quantitative assessment of deformation in this context and provides a new opportunity to inform predictive simulation of gas advection models in bentonite.

## Results

### Stress field monitoring in saturated bentonite

Stress measurements are conventionally made during bentonite swelling tests to assess the equilibration of the sample on hydration, usually in a constant volume or oedometer cell.^26^ monitored the stress field during gas injection through fully saturated Mx80 bentonite, constrained within a constant volume cell. They observed localised and abrupt changes in the stress field in multiple locations and attributed this to mechanical deformation of the clay following gas entry. Similar, localised disruption of the stress field was also observed during multiple gas injection tests at the large-scale gas injection test in the Äspö Underground Research Laboratory in Sweden^27^. Recent work has shown that analysis of first order fluctuations in the stress field contain information on deformation due to gas pressurisation^28–30^. In this study, we conduct a more extensive analysis (Section "Methods") using data from these 3 tests (Tests A, B and C) and a further test (D), involving a more heavily-instrumented stress field monitoring system (EURAD^31^). Findings are compared to well-studied natural deformation phenomena (Section "Methods"). Similar automatic algorithms are routinely used in seismology^32^ and have proven highly successful in the assessment of micromechanical deformation using acoustic emission data generated during rock deformation testing^33,34^. A brief overview of these experiments is given in Section "Methods". In all cases, Helium gas was used as a proxy for Hydrogen, so as to inhibit mass changes due to methanogenesis, as well as reducing safety complexities introduced by using this gas^35^. Helium represents a suitable proxy due to its similar atomic size and because the primary process under consideration is mechanical in nature it is reasonable to assume no significant differences in gas migration behaviour are likely.

Despite differing test histories, all three tests displayed (i) an initial phase of stress development (after the application of a constant water pressure), which stabilised before gas testing and (ii) a phase of gas pressure increase, resulting from injection, followed by gas pressure decline coinciding with major outflow downstream (gas breakthrough). In addition, all test samples remained fully saturated after testing, even after a substantial gas volume had passed through the sample (Section "Methods", Table 1).

**Table 1:** Sample information and geotechnical data.

Test number	Further details	Initial dry density (kg/m^3^)	Dimensions (mm) diameter(d) length (l)	Bulk saturation before testing (%)	Bulk saturation after testing (%)
A Mx80-10	Graham et al.^28^	1582	d = 60	98.6	101.3
			l = 120		
B Mx80-A1	Harrington et al.^29^	1560	d = 60	95	99
			l = 121		
C Mx80-A1	Harrington et al.^30^	Same sample as Test B	Same sample as Test B	Same sample as Test B	Same sample as Test B
D FPR-21–004	EURAD GAS^31^	1592	d = 60	93	Not available at time of publication
			l = 60		

**Figure 1:**
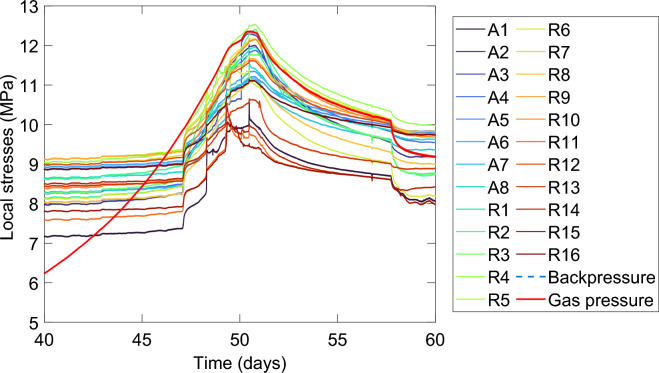
Example (Test D) showing disturbance of the stress field before and during gas breakthrough (sensors Axially orientated sensors A1-A8 and Radially orientated sensors R1-R16). See Methods (Fig. 8) for a schematic showing the sensor geometry used in this test.

During the gas pressurisation phase, monitored internal stresses remained unremarkable until gas pressure became close to the measured stress values. At this stage, small perturbations in the monitored stress field were detected in all experiments (Fig. 1). Several key observations were qualitatively apparent: (i) the magnitude of these stress perturbations (SP) differed at each sensor location, (ii) some perturbations occurred concurrently for some sensors, but (iii) not all sensors detected each perturbation event.

### Characteristics of geological fracture/faulting events

Fracturing and faulting phenomenon in rock have been intensively studied through a wide range of methodologies. In particular, seismological data provides information relating to the frequency, location, clustering and progression of both slip on tectonic faults and fracturing induced by volcanic activity. Studies of high frequency acoustic emissions generated during microfracturing of rock have also enabled the extensive quantitative analysis of deformation in the laboratory^36,37^. In this later case, the cumulative number of events with time has been used as a form of internal state variable representing the progressive weakening of rock on the approach to failure^36^.

Both earthquake and acoustic emission data have been widely shown to exhibit frequency-magnitude distributions that can be represented by power-law relations^38–41^. The Gutenberg-Richter relation describes the occurrence of earthquakes such that:

1 $$\mathit{log}N(M)=a-bM$$

where *N* is the number of earthquakes, of magnitude, *M*, and *a* and *b* are material constants.

**Figure 2:**
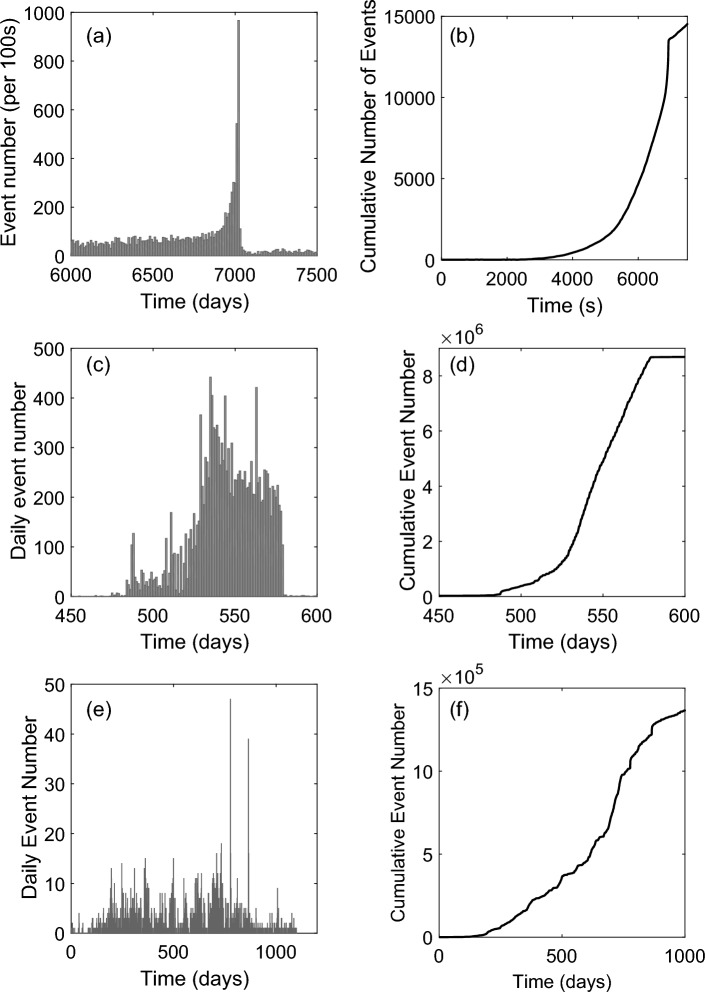
Example metrics from geological fractured systems, including: (**a**, **b**) acoustic emissions generated during fracture formation in granite, (**c**, **d**) detected earthquakes occurring in Turkey between 1997 and 2000 and (**e**, **f**) earthquakes recorded in the vicinity of Kilauea volcano, Hawaii, between 2017 and 2018. Data shown include: (i) the rate of event occurrence, (**a**, **c**, **e**) and (ii) the cumulative evolution of events with time, (**b**, **d**, **f**). Earthquake data was provided by the United States Geological Survey Earthquake catalog (https://earthquake.usgs.gov/earthquakes/search/).

Three datasets relating to energy release during geological fracturing were quantified using standard approaches (Section "Methods"), to allow comparison with gas injection data. Figure 2 shows the results for: (i) high frequency Acoustic Emissions (AE), generated by laboratory microfracturing of a granite sample subjected to triaxial compression^34^, (ii) tectonic earthquakes that occurred in Turkey, between 1997 and 2000 and (iii) earthquakes that occurred in the vicinity of Kilauea volcano, Hawaii, between 2017 and 2018. Event rates for laboratory rock fracture and volcanic eruption show distinct peak values (Fig. 2a,e), whereas the Turkey dataset, taken across a larger event population (Fig. 2c), exhibits multiple peaks. Figure 2 (b,d,f) also shows the cumulative number of events with time, during the progressive development/evolution of a fracture network. In all cases, this quantity shows a classic form, during acceleration to failure.

**Figure 3:**
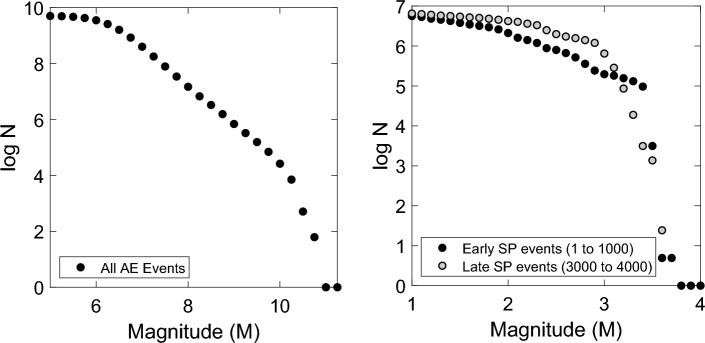
Cumulative frequency-magnitude distributions for Acoustic Emissions recorded during shear failure under triaxial compression in the Aue granite, after^34^ (left) and Stress Perturbation events recorded for 2 different test stages during gas injection into bentonite (Test D, right).

Finally, the frequency-magnitude distribution for the laboratory AE dataset is shown in Fig. 3 (left) and displays a classic form^36^. Such a relationship is generally considered to be evidence of fractal scaling, where a ‘b-value’ of *b* = *1* infers a truly scale invariant system^42–44^.

### Characteristics of stress perturbation events during gas flow

**Figure 4:**
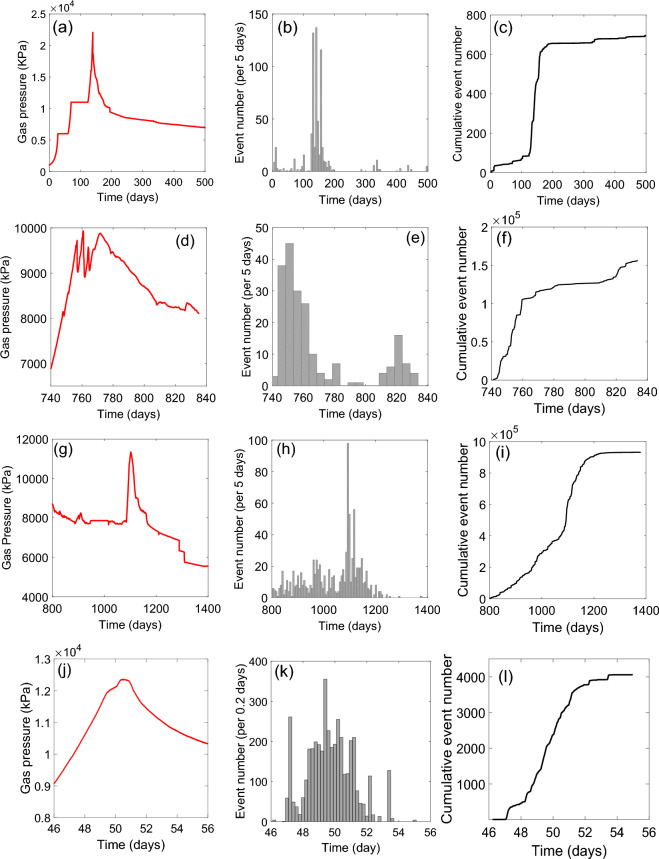
Example characteristics of stress perturbation events, taken from 4 gas injection experiments (Tests A, B, C and D) in bentonite clay. Data shown include: (i) gas pressure evolution (**a**, **d**, **g**, **j**), (ii) the rate of event occurrence, (**b**, **e**, **h**, **k**) and (iii) the cumulative evolution of events with time, (**c**, **f**, **i**, **l**).

Figure 4 shows qualitatively comparable metrics to those shown in Fig. 2, for the first order stress perturbation events detected during the 4 gas injection experiments (A, B, C and D). Evolution of gas pressure during injection is given for reference in red (Fig. 4a,d,g,j). Tests A, B and D were virgin gas injection events, whereas Test C was a continuation of Test B, where gas injection was repeated once a previous gas outflow event had ceased.

Despite the greatly differing origin of these sources, several similarities are apparent. Firstly, all 4 tests show an increase in the number of detected events that correlates with peak gas pressure and gas escape from the sample. Stress perturbation then reduces as gas pressure is allowed to decline.

Secondly, the cumulative event curves (Fig. 4c,f,i,l) have a similar form to those determined for the geological fracture-related events (Fig. 2b,d,f), demonstrating substantial disruption of the internal stress field in advance of gas escape, followed by relative quiescence and a plateau in activity. Furthermore, the greater sensor coverage and higher logging rate used in Test D meant the frequency-magnitude distributions of stress perturbation events could be assessed (Fig. 3, right). This shows a similar form to that observed for the AE data in Fig. 3. Sampling event data early on during gas injection and towards the final stages of the test (Fig. 3, right) indicates a change in slope, often detected during AE experiments^45^.

### Source analysis of stress perturbation events during gas flow

Many source analysis methodologies are used in seismology and acoustic emission studies to derive information about fracture mechanism, degree of slip and orientation^33,36,46–52^. These usually require knowledge of the source location in relation to the signal receiver. Whilst source locations and orientations are unknown for the gas test data considered in this study, an approach developed for the study of AE source mechanisms has been shown to produce valuable insight without this information^33,34^. The resulting ‘polarity’ metric has been shown to provide an indication of the degree of fracture dilatation (‘opening’) versus compression (‘closing’) behaviour that generated the detected signal.^33^ used this approach to classify events into three selected ranges: S-type events (shear) for a polarity between  − 0.25 and + 0.25, T-type (tensile) for pol <  − 0.25, or C-type (collapse) for pol >  + 0.25.

**Figure 5:**
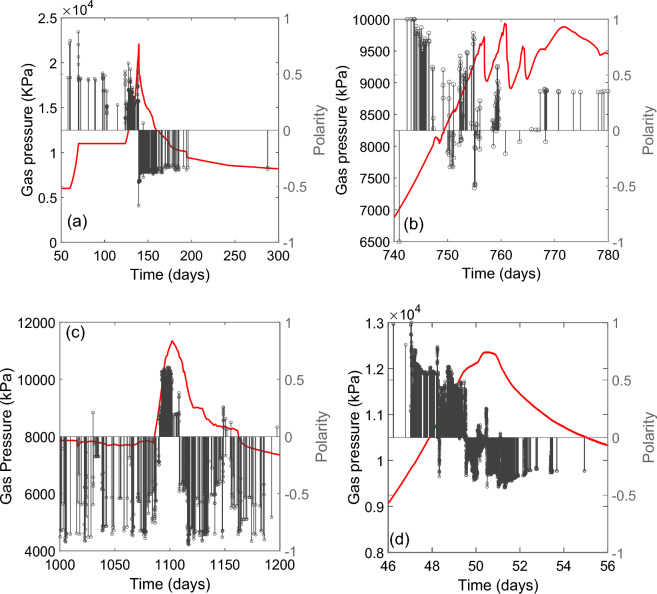
Gas pressure evolution (red line) and the associated polarity of detected stress perturbation events for Tests A, B, C and D (**a**–**d** respectively). Positive polarity values are associated with an element of ‘opening’ behaviour and negative events are dominated by ‘closing’. All tests show: (i) positive events occurring as gas pressure increases, (ii) negative events as gas pressure declines. Test A (**a**) shows a very distinct change from predominantly positive to negative events, which correlates with a rapid peak and decline in gas pressure. Test B (**b**) involved a complex phase of ‘sawtooth’ gas pressure evolution, characterised by episodic increase and decline in gas pressure (thought to relate to metastable opening and closure of gas pathways^29^). This behaviour correlates with positive polarity events during gas pressure increase and negative events when gas pressure declines. Test C (**c**) shows a repeat gas injection in a sample where gas flow has previously occurred. Event polarity is predominantly negative following the previous episode of pressurisation, but as gas pressure is allowed to increase, a substantial episode of positive events occurs until pressure declines and event polarity becomes negative. Test D (**d**) shows similar behaviour to Test A (**a**), but with a more apparent hiatus as gas pressure peaks, which correlates with a more complex phase of positive and negative events, before gas pressure decline begins.

Figure 5 shows polarity data calculated using this approach for the stress perturbation data. It should be noted that an opposing sign convention to^33^ has been used (predominantly opening behaviour results in a positive value with +1 representing a fully explosive source, whilst closing behaviour results in a negative value with −1 representing a fully implosive source). Fig. 5c relates to reinjection of gas after a previous gas breakthrough^30^. In all 4 tests, a period of positive polarity events was detected as gas pressure approached a peak, followed by an episode of negative polarity events afterwards.

### Spatial distribution of stress field perturbations

**Figure 6:**
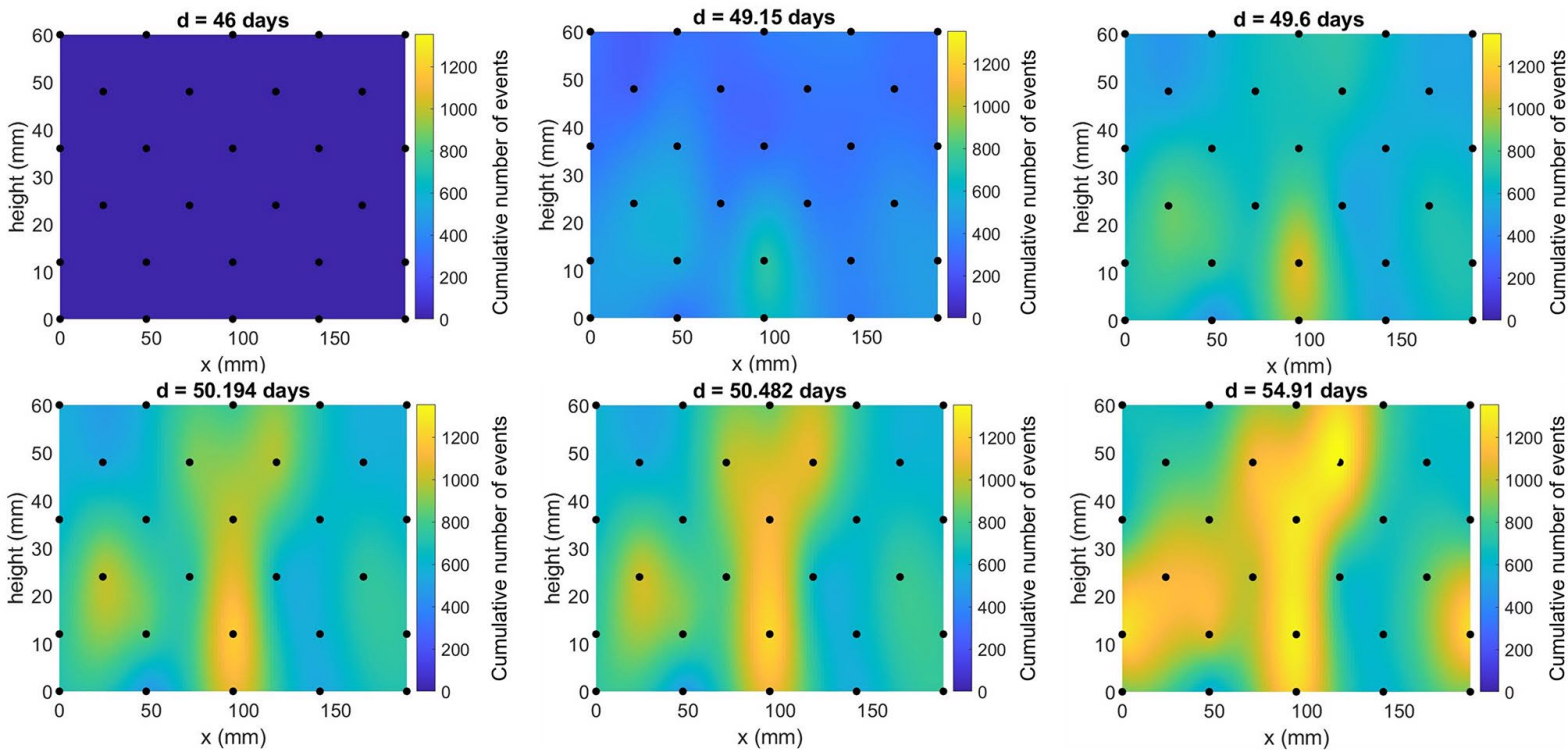
Total number of stress perturbation ‘events’ detected (cumulative number of events) by a given time in days. Values have no units and have been interpolated between all sensors (black circles) surrounding the sample. Values are plotted across the exterior surface of the sample, as a function of distance along cylinder axis (height) and distance along cylinder circumference (x) in (mm). The resulting distribution indicates a localised progression of ‘deformation’ during gas injection.

Whilst the cumulative number of unique stress perturbation events are shown in Fig. 4 (c,f,i,l), this data was derived from similar curves for each stress sensor. For test D, the new experimental set-up has a sufficient number of stress sensors (24) to interpolate both the raw stress data and the cumulative number of events detected at each sensor throughout the duration of gas entry and breakthrough (Fig. 6). It should be noted that the latter relates purely to the number of events detected at each sensor location, irrespective of source magnitude or distance from the sensor. Nevertheless, a notably localised distribution in event detections is apparent during testing, with the first events occurring at the same height as the gas injection point (a filter on a rod, embedded into the middle of the clay at a height = 12.5mm).

## Discussion

Multiple observations in the gas injection tests analysed in this study are consistent with gas flow occurring by deformation of the clay matrix, rather than displacement of water^10,29,30^. These include (i) the absence of sample desaturation after testing, (ii) an association between gas entry pressure and the total stress experienced by the clay, and (iii) the marked onset of first order perturbations in the stress field at this time, indicating hydromechanical coupling. Detailed analysis of these ‘events’, provide strong evidence to reinforce these conclusions (see Section "Results"), including (i) a rapid increase in detected events that correlates with peak gas pressure and gas escape from the sample and (ii) a rapid decrease in detected events that correlates with gas breakthrough. These findings are well-explained by disruption of the stress field, resulting from gas-driven deformation, but not by displacement flow. This highlights the powerful potential of this technique to discriminate the gas advection mechanism occurring in a specific experimental context.

Evolution of both the event rate and the cumulative number of events (Fig. 4), display a form which is similar to that for seismic events related to well-characterised fracture systems (Fig. 2). For virgin gas injection (Tests A, B and D; Fig. 4), both the rate of event occurrence and the cumulative evolution of events display a relatively rapid escalation to gas escape; behaviour most comparable to that shown for virgin laboratory fracture of granite and the Kilauea volcanic eruption. For a second gas injection episode, following a previous gas outflow event (Test C; Fig. 4), event clustering around gas breakthrough is still apparent, but there is a closer resemblance to the ongoing development of a pre-existing fault network, involving multiple event populations (Fig. 2).

Total cumulative event number is dependent not only on the degree of microdeformation occurrence, but also the detection limits of the system (number of sensors, rate of acquisition). With these datasets, it is, therefore, not appropriate to make direct comparisons of absolute values between both the earthquake/AE data and between the 4 gas tests presented, as these latter factors differ in some cases. However, this will be possible for future datasets where these factors are kept consistent.

In comparison, frequency-magnitude distributions provide an opportunity to examine a ‘sample’ of the microdeformation population. In this case, stress perturbation events display a similar trend to AE generated during microcracking of rock (Fig. 3). This distribution indicates 100’s or 1000’s of deformation events occurring within the clay in advance of gas breakthrough, spanning multiple scales. These findings are indicative of an evolving population of pathways with a range of sizes; the first insight into the 3D spatial distribution of this phenomenon under pressurised conditions. The change in slope over the test duration is commonly measured during rock fracture tests and suggests the potential to use this metric to provide further understanding on the evolution of the gas pathway network with time.

**Figure 7:**
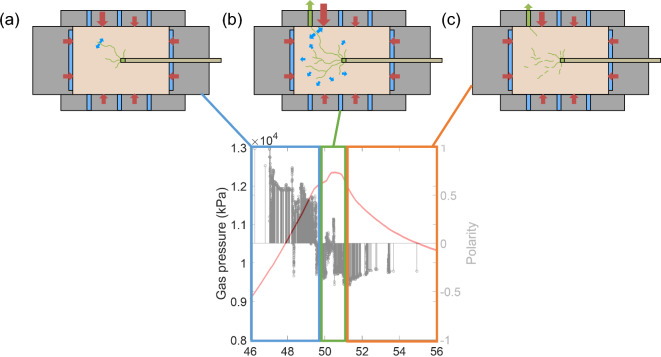
Modification of a conceptual diagram after^16^, demonstrating that the polarity metric provides quantitative evidence of the change in gas pathway behaviour during gas injection of bentonite. The following phases are identified: (**a**) gas has entered the clay and pathways beginning to open whilst gas pressure continues to rise, (**b**) gas is able to escape, pathways become unstable and may close and reopen as gas pressure peaks, (**c**) gas pressure declines sufficiently for gas pathways to continue to close.

The polarity source analysis approach provides further evidence of an association between clay deformation and gas flow (Fig. 5). Whilst less is known about the micromechanisms involved in this process than for AE in microfracture of rock, a positive polarity indicates creation of voidage, whilst a negative polarity suggests a collapse of voidage. A distinct correlation is apparent between increasing gas pressure and positive source polarity, whilst rapid gas pressure decay results in negative polarity sources. This can be explained by the opening of a gas pathway network in response to increasing gas pressures, followed by pathway closure once gas is able to escape the sample (Fig. 7). This is most apparent in the dataset generated by the simplest test history (Fig. 5a), where gas pumping ceased, allowing pressure decay.

For Test C (Fig. 5c), negative events predominate before and after peak gas pressure. This dataset, however, differs from the others since it follows a previous gas breakthrough event. These observations can be explained by an ongoing phase of self-sealing occurring in the clay, followed by a brief period of positive events when gas pressure was allowed to increase significantly, resulting in gas outflow, followed by a further phase of pathway closure^30^. These findings suggest the potential for this methodology to allow quantification of the degree of self-sealing after gas breakthrough.

Given that the polarity metric is derived entirely from stress field data, a distinct correlation with gas pressure represents strong evidence of mechanical coupling of gas flow in this context. These observations imply the creation of voidage enables gas migration, followed by the closure of this voidage as gas pressures decline.

Figure 6 provides insight into the spatial distribution of stress field disruption during gas flow in bentonite. It should be noted that Fig. 6 relates specifically to the number of events, but not their magnitude and reflects the cumulative degree of deformation detected at each sensor location. These findings demonstrate that the most significant stress field disruption in the early stages of gas entry occurs close to the injection rod (Section "Methods"), indicating a spatial association between gas injection and disruption of the stress field. Since disruption is then observed to grow in a relatively localised fashion towards the downstream end of the sample, without crossing the base of the sample (height = 0mm), it is possible to discount interfacial gas flow occurring between the walls of the vessel and the clay. Whilst this relates to the stress disturbance experienced around the exterior of the clay, this approach provides the first evidence of the spatial progression of deformation during gas migration through bentonite and hints at the potential for further refinement to allow true location analysis to be completed.

## Conclusion

Automated detection and analysis of first order stress field perturbations during gas advection in bentonite, irrefutably demonstrates mechanical disruption of the clay in this context. Comparison with well-established geological fracturing phenomena indicates that, for the material and boundary conditions examined, gas advection was enabled by deformation of the clay (pathway dilatancy). This is the first definitive technique for demonstrating this form of advection in the laboratory and provides a useful tool for discriminating between gas advection mechanisms in clay. Such an approach may also prove fruitful in a wider range of underground storage applications where the gas dominant advection mechanism of clay rich seals and shales needs to be assessed, or to examine the hydro-fracture process in shales.

This technique provides essential new information that can facilitate the development of numerical modelling of gas advection in clays. This includes: (i) the provision of internal state data that can be used to facilitate damage mechanics modelling, (ii) an initial understanding of the frequency-magnitude distribution of gas pathways, (iii) a metric quantifying the degree of fracture opening/closure during the gas pathway propagation and therefore self-sealing efficiency and (iv) insight into the spatial evolution of gas-driven stress field disruption under pressurised conditions. Frequency-magnitude data indicate the presence of a population of 100’s or 1000’s of gas pathways and is consistent with previous findings inferring chaotic behaviour in such systems^53^. In such cases, deterministic prediction may be less achievable. However, this methodology provides the quantification of both the degree and scaling of gas pathways necessary to test this possibility and to enhance the physical accuracy of gas pathway simulations. Improved representation of the gas flow processes will increase confidence in numerical predictions of gas pressure evolution and containment of radionuclides in geological disposal facilities, as well as providing insight into similar storage systems involving interaction between clay-rich geological materials and gas, from landfill clay liners to Carbon Capture Utillisation and Storage (CCUS).

Our findings also indicate that substantial information can be obtained by applying source analysis techniques to stress field data in the laboratory. Determination of event polarity also provides an important new tool to better understand self-sealing of fractures after gas pressure decay and to parameterise for this behaviour in numerical models. As with acoustic emissions in rock fracture, with a greater data sampling rate it may prove possible to invert for pathway location and orientation in 3D, allowing the true spatial mapping of gas pathway development. This additional data would prove invaluable, when combined with post-mortem analysis of post-test samples, to inform model development of gas advection in clays and ensure safe behaviour in geological disposal facilities.

## Methods

### Materials

Analysis is presented on datasets generated from gas flow conducted on samples of Mx-80 bentonite, a fine-grained, pre-compacted, sodium-rich clay (around 90% montmorillonite) from Wyoming. Blocks of pre-compacted Mx-80 bentonite were manufactured by Clay Technology AB (Lund, Sweden) by rapidly compacting bentonite granules in a mould under a one dimensionally applied stress^54^. Geotechnical properties are given in Table 1.

### Gas injection experiments

**Figure 8:**
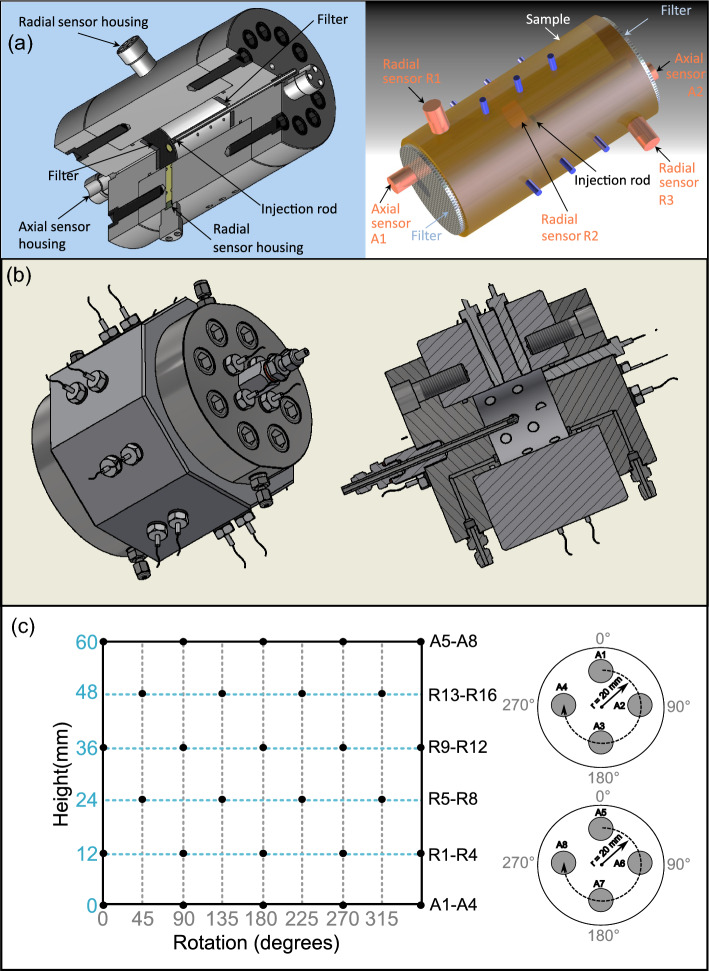
Test apparatus schematics and sensor locations. (**a**) Tests A-C: (left) Cut-away schematic of the apparatus, (right) Sensor locations in relation to the sample surface, within the test vessel. (**b**) Test D: Cut-away schematic of the test apparatus. (**c**) Test D: Sensor location map, plotted as a projection of the cylindrical sample exterior surface and the two sample ends. Sensors are shown as black and grey circles, respectively.

Data was utilised from 4 gas injection experiments: A, B, C and D. Sample geotechnical characteristics and associated publications detailing test methodologies are given in Table 1. Test stages varied, but in all cases: (i) the clay was near fully saturated with water before gas testing, (ii) the clay was constrained within a rigid stainless-steel vessel, enforcing a constant volume boundary condition which resulted in the development of an internal stress due to swelling of the clay, (iii) gas was injected through a central rod, inserted into the interior of the cylindrical test sample, (iv) local total stress development during saturation and gas injection was monitored at either 2 axial and 3 radial locations (Tests A-C; Fig. 8a left and right), or 8 axial and 16 radial locations (Test D; Fig. 8b and c). An array of filters provided the possibility for gas to escape from at least one filter during testing, though the geometry of these varied.

In all cases, the bentonite was allowed to hydrate at a constant applied water pressure. During hydration and swelling under a constant volume boundary condition, all samples experienced development of internal stresses, which can be expressed as a tensor quantity, ${\sigma }_{ij}$;

2 $${\sigma }_{ij}={\sigma }_{ij}^{e}+\alpha {\delta }_{ij}{P}_{p}$$

where ${\sigma }_{ij}^{e}$ is the effective stress resulting from the opposing pore pressure, ${P}_{p}$, the Biot parameter, $\alpha$, and the Kroekner delta, ${\delta }_{ij}$. For simplicity, this is commonly reduced to the consideration of three perpendicular principal stresses. Stress sensors were calibrated in advance of all 4 tests by applying a series of incremental pressure steps and monitoring output compared to a pressure calibrator.

The clay was allowed to equilibrate until changes in stress development and inflow of water were considered negligible. Helium gas was injected at a constant flow rate. Samples were maintained at constant downstream water pressure during gas injection. Gas breakthrough across the sample was inferred by monitoring the fluid volume in the downstream pump maintaining this pressure. Stress analysis was conducted for all four gas injection experiments. Tests A, B and D were performed on intact clay, which had never experienced a previous gas injection test, whilst Test C^30^ was a repeat gas injection test carried out following a previous injection test on the same sample (Test B).

### Earthquake and acoustic emission analysis

Three datasets were analysed: (i) laboratory acoustic emission data generated during the triaxial deformation of a granite sample, (ii) earthquake data for events in Turkey and (iii) earthquake data for events at Kilauea volcano, Hawaii.

The acoustic emission data was collected during shear localisation of granite^34^. Earthquake data were collected from the USGS Earthquake catalogue (timeframe and area given in Table 2). Timeframe for the Kilauea data includes the run-up to and progress of the 2018 rift eruption and summit collapse^55^. The Turkey dataset spanned a timeframe during which significant events occurred, including the Ceyhan (magnitude 6.2), Izmit (magnitude 7.6) and Düzce (magnitude 7.2) earthquakes^56–58^. Only events with a minimum magnitude of 2.5 were included.

**Table 2:** Selection criteria for earthquake data.

Dataset	Start date	End date	Latitudes	Longitudes
Turkey	01/01/1997	31/12/2000	35.914, 42.055	26.088, 44.898
Kilauea volcano	01/01/2017	31/12/2018	19.08, 19.534	− 155.525, − 154.763

The rate of detected events was considered by assessing the number of events detected within a given time window (Fig. 2a,c,e). To allow for the different timescales involved, window durations of 1 day and 100s were used for the earthquake data and the acoustic emission data, respectively. The cumulative frequency magnitude for the AE dataset was calculated using Eq. (1).

### Stress analysis

**Figure 9:**
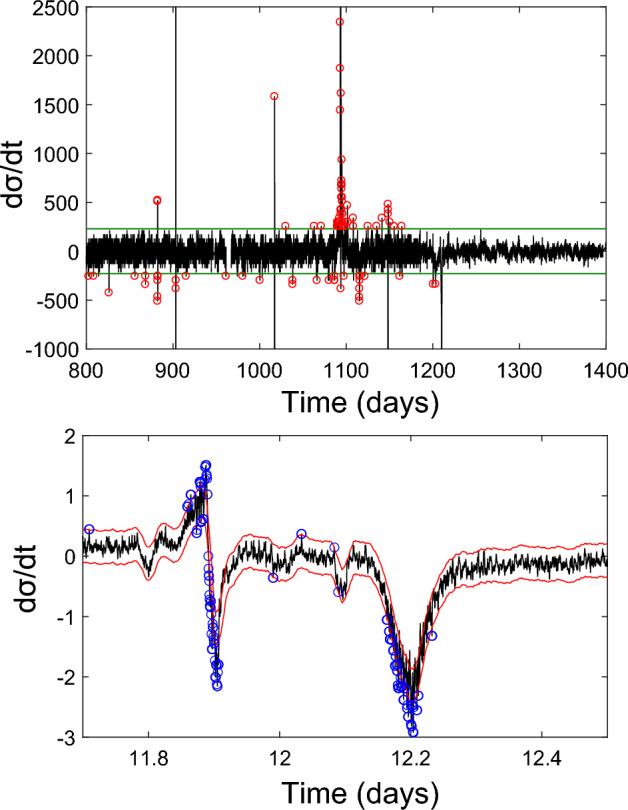
Example event picks using: (top) linear noise thresholds (applied to Tests A, B, C) and (bottom) a rolling window threshold (applied to the higher resolution data from Test D).

First order perturbations in the stress field were detected, or ‘picked’, using an automated algorithm designed to assess their presence above background levels. Any initial offset was removed and the first derivative found, with respect to time, for each stress sensor (Fig. 9). For data from Tests A, B and C, a linear upper and lower noise threshold were set (Fig. 9), based on the standard deviation (s.d.) of a manually selected ‘baseline’ section of the dataset (consisting of 550 data-points), before gas entry. With the greater sensor coverage and higher logging rates used in Test D a more sophisticated approach could be applied, using a variable noise threshold, also based on 2 times the standard deviation (s.d.) for a rolling window. Individual signal peaks and troughs occurring above and below the chosen noise thresholds were then found using a rolling window with a quarter-length overlap. The resulting ‘picks’ were then examined and thresholds of 3 × s.d. were found to provide satisfactory results for the exclusion of data below these values. Whilst further events may remain undetected below these thresholds, it was considered better to exclude a few minor events than add many additional ‘false’ events, resulting from the noise thresholds being set too low.

Testing was conducted under temperature-controlled conditions, but thermocouple data showed a few minor excursions in laboratory temperature were observed to coincide with stress perturbation events. The same picking algorithm was therefore used to find the time of all thermal excursion events (above a noise threshold). Stress perturbations found to occur within 1 h of a temperature excursion were then removed from the catalogue, except for Tests A and D, where thermocouple data was not available. Selected stress perturbation ‘events’ were collated into a catalogue of detection times and magnitudes (peak or trough dσ/dt). Where detection times were found to correlate across multiple sensors, they were assumed to derive from the same event and counted only once in calculated event rate and cumulative event number curves, following the same approach as described in Section "Earthquake and acoustic emission analysis".

The cumulative frequency-magnitude distribution for Test D was calculated using the same approach as for the acoustic emission data, except that (following the approach given by^36^ and^59^) the magnitude, M, was calculated from the peak amplitude, ${A}_{peak}$, of each detected event, such that:

3 $$M=\mathrm{log} ({A}_{peak})$$

### Polarity source analysis method

A simple method was used for estimating the predominant source behaviour, based on a methodology applied to acoustic emissions by^33^. The first motion amplitude, Ai, at k sensors, is used to find an average polarity for each event, according to:

4 $$pol=\frac{1}{k}\sum_{i=1}^{k}sign({A}_{i})$$

This provides an estimate of net polarity of volume change at the location of the source. Event magnitude data were used in this study to provide normalised polarity values for stress perturbation events between  − 1 and + 1.

## Data Availability

The datasets used in this article and generated by the British Geological Survey are all published elsewhere and these studies are referred to in the text. These datasets are available from the corresponding author on reasonable request. The seismic data from Turkey and Kilauea volcano are available from the United States Geological Survey Earthquake Catalog (https://earthquake.usgs.gov/earthquakes/search/).
